# Prediction of non-dipper blood pressure pattern in Chinese patients with hypertension using a nomogram model

**DOI:** 10.3389/fphys.2024.1309212

**Published:** 2024-07-24

**Authors:** Dandan Sun, Zhihua Li, Guomei Xu, Jing Xue, Wenqing Wang, Ping Yin, Meijuan Wang, Miaomiao Shang, Li Guo, Qian Cui, Yuchuan Dai, Ran Zhang, Xueting Wang, Dongmei Song

**Affiliations:** Department of Cardiology, Affiliated Hospital of Jining Medical University, Jining, China

**Keywords:** hypertension, nomogram, dipper, non-dipper, blood pressure pattern

## Abstract

Non-dipper blood pressure has been shown to affect cardiovascular outcomes and cognitive function in patients with hypertension. Although some studies have explored the influencing factors of non-dipper blood pressure, there is still relatively little research on constructing a prediction model. This study aimed to develop and validate a simple and practical nomogram prediction model and explore relevant elements that could affect the dipper blood pressure relationship in patients with hypertension. A convenient sampling method was used to select 356 inpatients with hypertension who visited the Affiliated Hospital of Jining Medical College from January 2022 to September 2022. All patients were randomly assigned to the training cohort (75%, n = 267) and the validation cohort (25%, n = 89). Univariate and multivariate logistic regression were utilized to identify influencing factors. The nomogram was developed and evaluated based on the receiver operating characteristic (ROC) curve, the area under the ROC curve (AUC), and decision curve analyses. The optimal cutoff values for the prevalence of dipper blood pressure were estimated. The nomogram was established using six variables, including age, sex, hemoglobin (Hb), estimated glomerular filtration rate (eGFR), ejection fraction (EF), and heart rate. The AUC was 0.860 in the training cohort. The cutoff values for optimally predicting the prevalence of dipper blood pressure were 41.50 years, 151.00 g/L, 117.53 mL/min/1.73 m^2^, 64.50%, and 75 beats per minute for age, Hb, eGFR, ejection fraction, and heart rate, respectively. In summary, our nomogram can be used as a simple, plausible, affordable, and widely implementable tool to predict the blood pressure pattern of Chinese patients with hypertension.

## Introduction

Worldwide, the prevalence of hypertension is steadily increasing and has become the leading cause of preventable death (Jones et al., 2020; Murray and Aravkin, 2020). According to a large-scale nationwide survey, the crude prevalence rate of hypertension in the Chinese population is 23.2% (≈244.5 million), and that of pre-hypertension is 41.3% (≈435.3 million), with both rates rising with age (Wang et al., 2018). In healthy individuals, blood pressure follows a regular circadian rhythm, with two peaks during the day (6:00–8:00 and 4:00–16:00) and a 10%–20% drop at night (Smolensky et al., 2017). This regular circadian pattern is referred to as dipper blood pressure. However, some patients with hypertension experience disrupted circadian rhythms. For these patients, nighttime blood pressure does not decrease significantly compared to daytime blood pressure, or it may even be higher at night; this condition is termed non-dipper blood pressure (Akasaki and Ohishi, 2014; Wójcik et al., 2022). In recent years, the widespread use of 24-h ambulatory blood pressure monitoring in clinical practice has increased attention to the clinical significance of blood pressure patterns (Guirguis-Blake et al., 2021; Chia et al., 2022). Non-dipper blood pressure has been shown to negatively impact cardiovascular outcomes and cognitive function in patients with hypertension (Hjortkjær et al., 2022; Daniela et al., 2023), and it is associated with multiple organ function injuries (Akyüz and Işık, 2022). However, not all patients have access to 24-h ambulatory blood pressure monitoring. Therefore, developing a simple and rapid method for predicting patients’ blood pressure patterns is significant. Based on this premise, this study aimed to identify the factors influencing blood pressure patterns in patients with hypertension and develop a practical nomogram prediction model.

## Methods

### Participants

**FIGURE 1:**
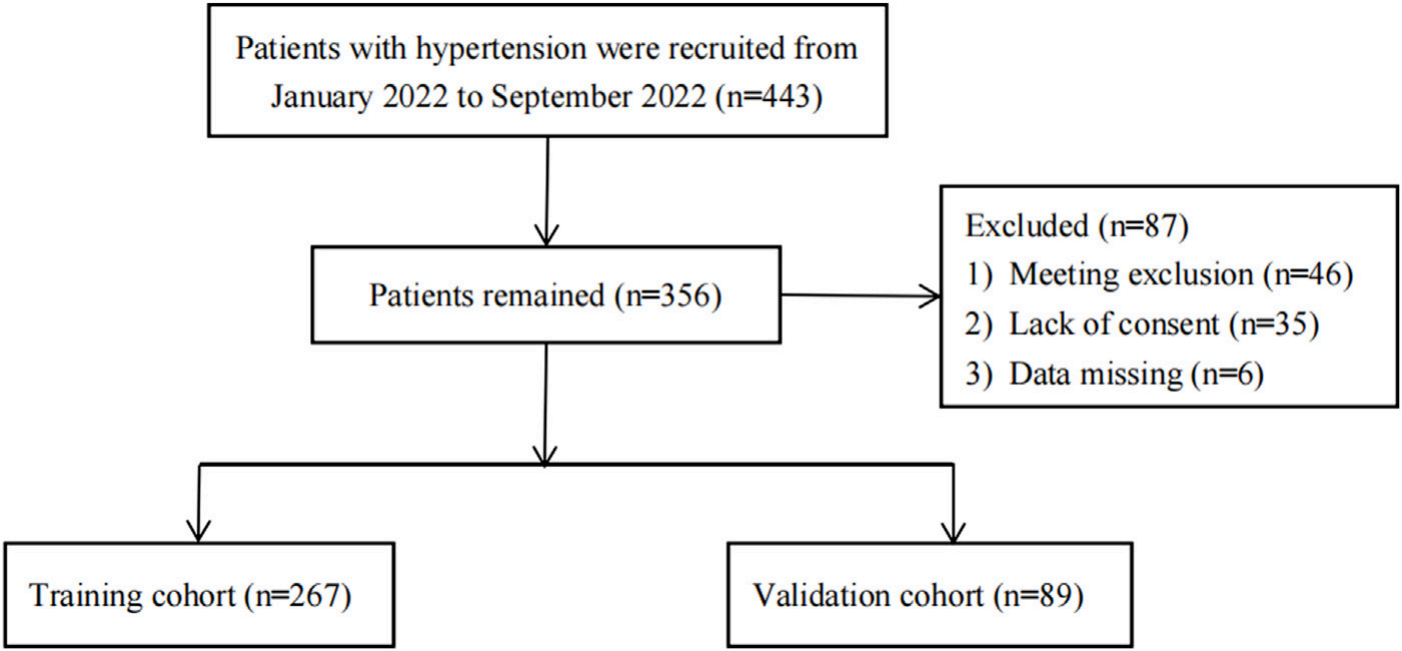
Flowchart of patients.

This cross-sectional study employed convenience sampling to select 356 patients admitted to the Hypertension Ward of the Department of Cardiology, Affiliated Hospital of Jining Medical University, from January 2022 to September 2022. The patient inclusion flowchart is depicted in Figure 1. The inclusion criteria were: 1) Patients diagnosed with hypertension according to the Chinese Guidelines for the Prevention and Treatment of Hypertension (2018 Revised Edition). Hypertension was diagnosed by measuring blood pressure thrice on different days without anti-hypertensive medication, with systolic pressure ≥140 mmHg, diastolic pressure ≥90 mmHg, or both. Patients with a history of hypertension were diagnosed even if their blood pressure was <140/90 mmHg while on anti-hypertensive medications. 2) Participants were at least 18 years old. 3) Patients had good audiovisual abilities and could effectively complete the questionnaire. Exclusion criteria were: 1) Patients in the acute stage of inflammation; 2) Patients with acute heart failure; 3) Patients with atrial fibrillation; 4) Pregnant patients; 5) Patients with tumors or other terminal-stage diseases. All participants provided informed consent, and the study was reviewed and approved by the Ethics Committee of the Affiliated Hospital of Jining Medical University (NO. 2023-04-C016).

### Data collection

Uniform training was conducted for all team researchers to ensure the quality and consistency of data collection. Upon admission, general information, including age, sex, medical history, height, and weight, was collected, and the body mass index (BMI) was calculated as weight (kg)/(height in m)^2^ (m^2^). All participants fasted for solids and liquids after 22:00 on the admission day, and fasting blood samples were collected the following morning. The collected data included hemoglobin (Hb), creatinine, urea, uric acid, triglycerides (TG), total cholesterol (TC), high-density lipoprotein (HDL-C), low-density lipoprotein (LDL-C), homocysteine (HCY) platelet-to-lymphocyte ratio (PLR), neutrophil-to-lymphocyte ratio (NLR), and red cell distribution width (RDW). The estimated glomerular filtration rate (eGFR) was calculated using the following formula: eGFR (mL/min/1.73 m^2^) = 141×min (Cr/k,1)^a^×max (Cr/k,1)^−1.209^ × 0.993^Age^×1.018 (female) k = 0.7 (female), 0.9 (male); a = -0.329 (female), −0.411(male). Two researchers cross-checked the data before entering it into the database. An experienced sonographer determined the ejection fraction of the heart (EF). The patients’ medication history was collected over the previous 30 days, categorizing medications into ARNI, *β*-block, CCB, diuretic, ARB/ACEI, and arotinolol. Blood pressure was measured in a quiet, warm environment using an OMRON (HBP-1320) blood pressure monitor (OMRON, Kyoto, Japan). The same nurse measured the patient’s blood pressure in the supine position after 1 and 3 min of standing. According to the 1996 American Society for Autonomic Neuroscience, orthostatic hypotension (OH) is defined as a decrease in systolic blood pressure >20 mmHg or a decrease in diastolic blood pressure >10 mmHg or both within 3 min of moving from a supine to a standing position (H, 1996).

Patients were fitted with a 24-h ambulatory blood pressure monitor (Version: Standley W-BPA), which recorded their blood pressure changes over 24 h. Blood pressure was measured every 30 min during the day (6:00–22:00) and every hour at night (22:00–6:00). Data with an effective rate of >85% were considered valid measurements. The 24-h mean systolic and diastolic blood pressures were recorded. The mean arterial pressure (MAP) for daytime and nighttime were calculated. The MAP was calculated using the formula: MAP = (systolic blood pressure +2× diastolic blood pressure)/3. The nocturnal blood pressure drop rate was calculated as (daytime MAP - nighttime MAP)/daytime MAP. If the nocturnal drop rate of blood pressure was more than 10%, the patient’s blood pressure pattern was recorded as a dipper blood pressure. If the nocturnal blood pressure drop rate was less than 10% or the nocturnal blood pressure increased, the pattern was recorded as non-dipper blood pressure (Koike et al., 2023).

Pittsburgh Sleep Quality Index (PSQI): Subjective sleep quality and sleep disturbances over the past month were measured using the PSQI (Buysse et al., 1989). This self-rating scale consists of 19 items divided into seven components: subjective sleep quality, sleep latency, sleep duration, sleep efficiency, sleep disturbances, use of sleeping medication, and daytime dysfunction. The total PSQI score ranges from 0 to 21, with higher scores indicating poorer sleep quality. A cumulative score above seven was indicative of inadequate sleep. This scale has been validated in the Chinese population, showing a sensitivity and specificity of 98.3% and 90.2%, respectively. The total Cronbach’s α coefficient was 0.845, and the test-retest reliability was 0.994.

### Statistical methods

Participant characteristics, stratified by training and validation cohorts, were presented as means (standard deviations) or medians (interquartile ranges) for continuous variables, and as numbers and percentages for categorical variables. One-way analysis of variance (ANOVA) and Kruskal–Wallis tests were used to analyze differences between groups for normally distributed and skewed continuous variables, respectively. The chi-squared test was conducted for analyzing categorical variables (Table 1).

**TABLE 1:** Characteristics of the training and validation groups.

Characteristic	Training group (n = 267)	Validation group (n = 89)	*p*-Value
Age, years	47.62 ± 12.48	47.58 ± 12.53	0.979
Sex, n (%)			0.885
Male	159 (59.55%)	54 (60.67%)	
Female	108 (40.45%)	35 (39.33%)	
CHD, n (%)			0.764
0	228 (85.39%)	77 (86.51%)	
1	39 (14.61%)	12 (13.49%)	
Heart failure, n (%)			0.856
0	263 (98.50%)	88 (98.88%)	
1	4 (1.50%)	1 (1.12%)	
Diabetes, n (%)			0.890
0	210 (78.65%)	71 (79.78%)	
1	57 (21.35%)	18 (20.22%)	
BMI, kg/m^2^	26.28 ± 4.06	26.19 ± 3.98	0.856
Heart rate, times per minutes	75.96 ± 13.83	76.12 ± 13.11	0.904
Hb, g/L	139.71 ± 15.22	139.34 ± 15.01	0.842
PLR	139.93 ± 45.49	131.72 ± 36.92	0.144
NLR	0.36 ± 0.15	0.33 ± 0.99	0.100
RDW,fl	0.12 ± 0.01	0.12 ± 0.01	0.862
Creatinine, µmol/L	67.41 ± 27.18	69.20 ± 31.60	0.611
eGFR, ml/min/1.73m^2^	105.19 ± 18.99	104.03 ± 21.27	0.633
Urea, mmol/L	5.49 ± 1.88	5.51 ± 1.79	0.930
Uric acid, µmol/L	339.66 ± 104.95	336.40 ± 103.21	0.799
TG, mmol/L	1.78 ± 1.34	1.79 ± 1.31	0.951
TC, mmol/L	4.74 ± 1.31	4.76 ± 1.33	0.901
HDLC, mmol/L	1.24 ± 0.30	1.24 ± 0.28	0.994
LDLC, mmol/L	3.00 ± 1.02	3.01 ± 1.01	0.936
HCY, µmol/L	11.06 ± 4.19	10.89 ± 4.12	0.739
EF, %	61.80 ± 3.53	60.96 ± 3.66	0.054
Medicine			
ARNI	129 (48.3%)	51 (57.3%)	0.089
β-block	79 (29.6%)	21 (23.6%)	0.170
CCB	122 (45.7%)	33 (37.1%)	0.097
Diuretic	27 (10.1%)	4 (4.4%)	0.069
ARB/ACEI	42 (15.7%)	8 (9.0%)	0.076
Arotinolol	18 (6.7%)	7 (7.9%)	0.439
PSQI	7.70 ± 3.71	7.72 ± 3.74	0.965
OH	45 (16.9%)	15 (16.7%)	0.550
Daytime SBP, mmHg	132.94 ± 13.45	133.17 ± 13.53	0.892
Nighttime SBP, mmHg	127.06 ± 15.94	127.49 ± 16.27	0.826
Daytime DBP, mmHg	86.50 ± 10.77	86.47 ± 10.73	0.977
Nighttime DBP, mmHg	82.58 ± 12.05	82.78 ± 12.11	0.893
24-MEANSBP, mmHg	131.83 ± 13.14	132.03 ± 13.10	0.901
24-MEANDBP, mmHg	85.62 ± 10.75	85.70 ± 10.81	0.952

CHD, coronary heart disease; BMI, body mass index; Hb, hemoglobin; PLR, platelet-to-lymphocyte ratio; NLR, neutrophil-to-lymphocyte ratio; RDW, red cell distribution width; eGFR, estimated glomerular filtration rate; TG, triglyceride; TC, total cholesterol; HDL-C, high density lipoprotein cholesterol; LDL-C, low density lipoprotein cholesterol; Hcy, homocysteine; EF, ejection fraction; PSQI, pittsburgh sleep quality index; OH, orthostatic hypotension; Daytime SBP, daytime systolic blood pressure; Nighttime SBP, nighttime systolic blood pressure; Daytime DBP, daytime diastole blood pressure; Nighttime DBP, nighttime diastole blood pressure; 24-h mean SBP, 24-h mean systolic blood pressure; 24-h mean DBP, 24-h mean diastole blood pressure.

Table 2 presents baseline characteristics of the training cohort, stratified by the prevalence of non-dipper blood pressure. Influencing factors for non-dipper blood pressure were analyzed using univariate and multivariate logistic regression analysis with generalized estimating equations (Tables 2, 3). Results are presented as *β* coefficients and odds ratios (OR) with associated 95% confidence intervals (95% CI).

**TABLE 2:** Baseline characteristics according to the prevalence of non-dipper blood pressure and the univariate logistic regression analysis in the training group (n = 267).

Characteristic	Prevelance of non-dipper BP		Univariate Logistic regression analysis	
	Non-dipper (n = 198)	Dipper BP (n = 69)	OR	*P*
Age, year	50.45 ± 11.48	39.48 ± 11.67	1.09 (1.06, 1.12)	<0.001
Sex, n (%)			1.79 (1.08, 2.97)	0.024
Male	111 (56.06%)	48 (69.57%)		
Female	87 (43.94%)	21 (30.43%)		
CHD, n (%)			2.10 (0.95, 4.65)	0.067
0	165 (83.33%)	63 (91.30%)		
1	33 (16.67%)	6 (8.70%)		
Heart failure, n (%)			0.18 (−0.10, 0.45)	0.771
0	195 (98.48%)	68 (98.55%)		
1	3 (1.52%)	1 (1.45%)		
Diabetes, n (%)			1.40 (0.76, 2.57)	0.283
0	153 (77.27%)	57 (82.61%)		
1	45 (22.73%)	12 (17.39%)		
BMI, kg/m^2^	25.55 ± 3.54	28.36 ± 4.64	0.83 (0.78, 0.89)	<0.001
Heart rate, times per minutes	73.65 ± 13.50	83.04 ± 12.27	0.95 (0.93, 0.97)	<0.001
Hb, g/L	136.93 ± 14.57	149.35 ± 13.04	0.93 (0.90, 0.95)	<0.001
PLR	140.08 ± 47.19	137.82 ± 27.13	1.00 (0.99,1.01)	0.818
NLR	0.37 ± 0.16	0.33 ± 0.12	0.24 (0.01, 4.70)	0.354
RDW,fl	0.12 ± 0.01	0.12 ± 0.01	inf. (0.00, inf.)	0.276
Creatinine, µmol/L	66.92 ± 26.28	60.84 ± 11.56	0.99 (0.97, 1.01)	0.174
eGFR, ml/min/1.73m^2^	100.43 ± 19.42	116.48 ± 13.37	1.07 (1.04, 1.11)	<0.001
Urea, mmol/L	5.67 ± 2.00	4.84 ± 1.11	1.49 (1.17, 1.90)	0.001
Uric acid, µmol/L	334.72 ± 101.20	357.56 ± 114.39	1.00 (1.00, 1.00)	0.122
TG, mmol/L	1.76 ± 1.42	1.84 ± 0.94	0.96 (0.78, 1.16)	0.648
TC, mmol/L	4.83 ± 1.34	4.45 ± 1.10	1.27 (1.01, 1.59)	0.040
HDLC, mmol/L	1.28 ± 0.32	1.12 ± 0.14	12.45 (3.04, 51.07)	<0.001
LDLC, mmol/L	3.04 ± 1.04	2.89 ± 0.94	1.15 (0.87, 1.52)	0.324
HCY, µmol/L	10.86 ± 3.33	11.67 ± 5.99	0.96 (0.91, 1.01)	0.127
EF, %	61.25 ± 3.45	63.58 ± 3.10	0.78 (0.70, 0.86)	<0.001
Medicine				
ARNI	98 (49.5%)	31 (44.9%)	0.83 (0.48, 1.44)	0.304
β-block	60 (30.3%)	19 (27.5%)	0.87 (0.47, 1.61)	0.393
CCB	88 (44.4%)	35 (50.7%)	1.29 (0.74, 2.23)	0.223
Diuretic	18 (9.1%)	9 (13.0%)	0.65 (0.31, 1.88)	0.122
ARB/ACEI	29 (14.6%)	14 (20.3%)	1.48 (0.73, 3.01)	0.181
Arotinolol	15 (7.6%)	4 (5.8%)	0.75 (0.24, 2.34)	0.427
PSQI	8.18 ± 3.91	6.30 ± 2.54	1.19 (1.10, 1.30)	<0.001
OH	36 (18.2%)	9 (13.0%)	0.68 (0.31, 1.49)	0.216
Daytime SBP, mmHg	131.74 ± 13.24	136.42 ± 13.73	1.03 (1.01, 1.05)	0.014
Nighttime SBP, mmHg	130.58 ± 15.80	116.91 ± 12.48	0.93 (0.91, 0.96)	<0.001
Daytime DBP, mmHg	86.11 ± 10.51	87.84 ± 11.37	1.02 (0.99, 1.05)	0.248
Nighttime DBP, mmHg	85.04 ± 11.31	75.80 ± 11.77	0.93 (0.91, 0.96)	<0.001
24-MEANSBP, mmHg	131.53 ± 13.16	132.70 ± 12.89	0.99 (0.98, 1.01)	0.462
24-MEANDBP, mmHg	85.71 ± 10.40	85.35 ± 11.58	1.00 (0.98, 1.03)	0.778

CHD, coronary heart disease; BMI, body mass index; Hb, hemoglobin; PLR, platelet-to-lymphocyte ratio; NLR, neutrophil-to-lymphocyte ratio; RDW, red cell distribution width; eGFR, estimated glomerular filtration rate; TG, triglyceride; TC, total cholesterol; HDL-C, high density lipoprotein cholesterol; LDL-C, low density lipoprotein cholesterol; Hcy, homocysteine; EF, ejection fraction; PSQI, pittsburgh sleep quality index; OH, orthostatic hypotension; Daytime SBP, daytime systolic blood pressure; Nighttime SBP, nighttime systolic blood pressure; Daytime DBP, daytime diastole blood pressure; Nighttime DBP, nighttime diastole blood pressure; 24-h mean SBP, 24-h mean systolic blood pressure; 24-h mean DBP, 24-h mean diastole blood pressure.

**TABLE 3:** Multivariate logistic regression analysis for related factors associated non-dipper blood pressure in the training cohort (n = 267).

Variable	*β*	*SE*	Wald *χ* ^2^	P	*OR*
Age, year	0.116	0.038	9.442	0.002	1.123
Sex, n (%)				<0.001	0.005
Male	−5.372	0.972	30.579		
Female	Ref				
Hb, g/L	−0.201	0.035	33.460	<0.001	0.818
eGFR, ml/min/1.73m^2^	−0.051	0.023	4.736	0.030	0.951
EF, %	−0.496	0.130	14.540	<0.001	0.609
Heart rate, times per minutes	−0.050	0.021	5.657	0.017	0.951

Hb, hemoglobin; eGFR, estimated glomerular filtration rate; EF, ejection fraction.

**FIGURE 2:**
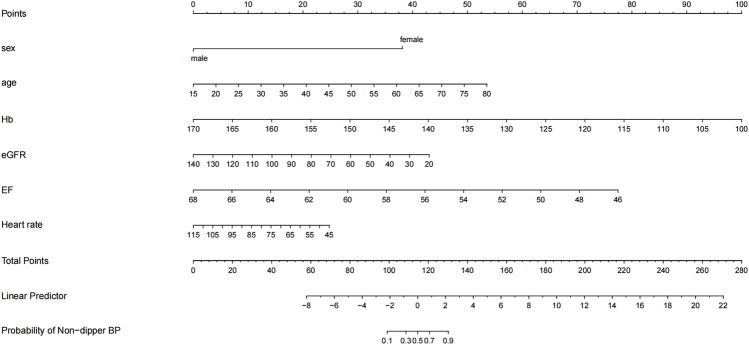
Nomogram to predict non-dipper blood pressure pattern of patients with hypertension.

**FIGURE 3:**
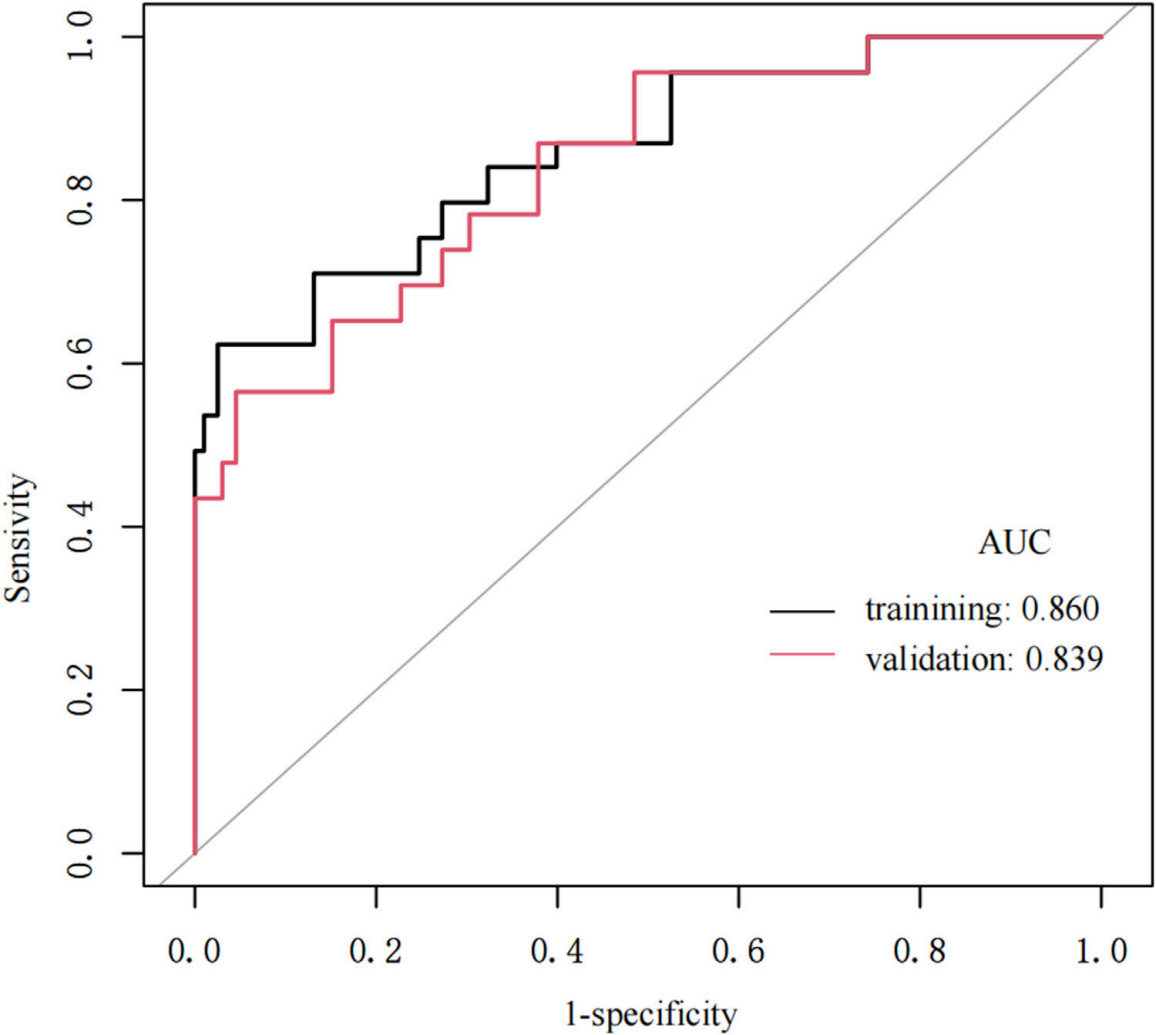
The ROC curves of the nomogram for Non-dipper BP of hypertension patients in the training cohort and validation cohort.

**FIGURE 4:**
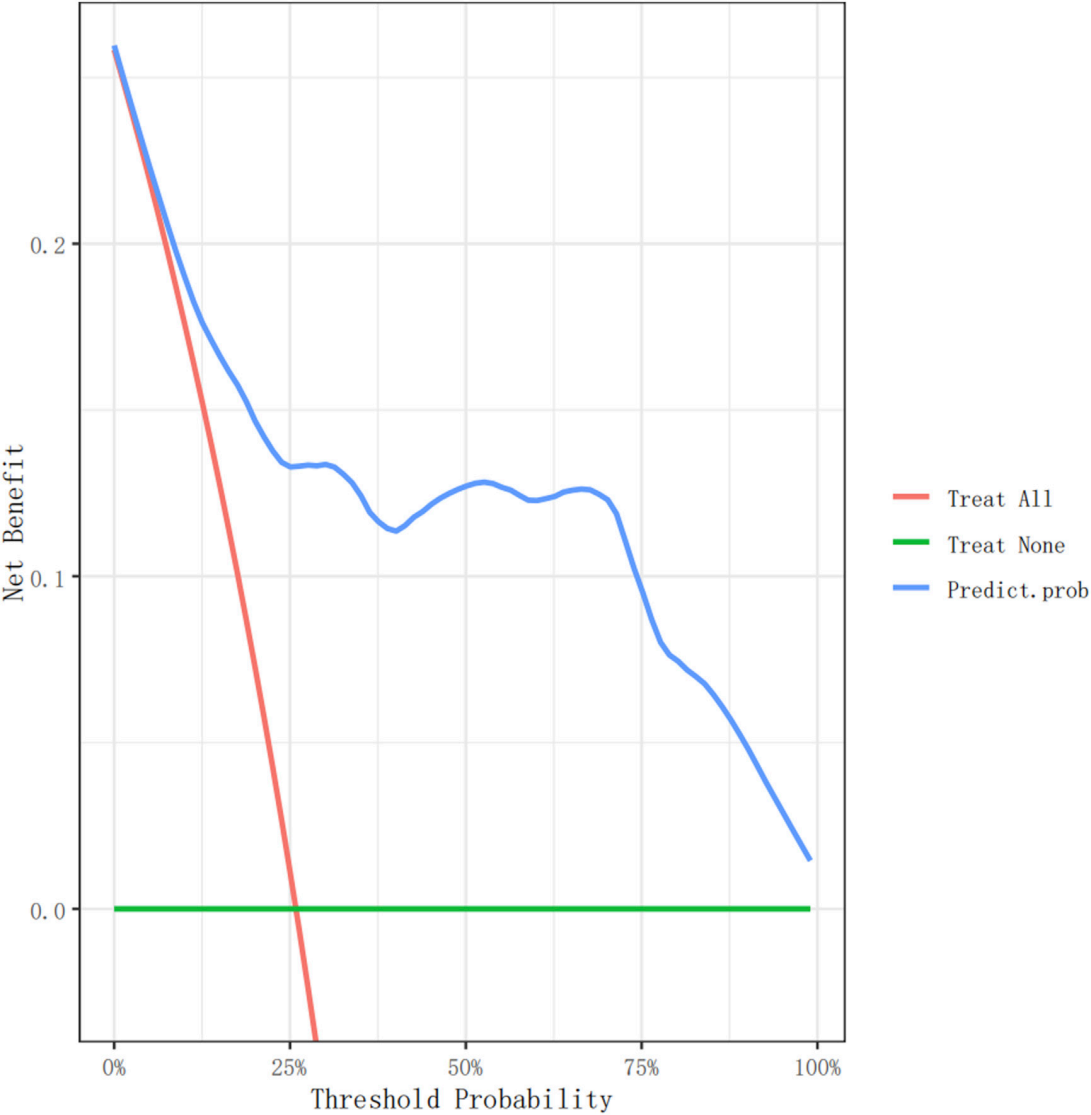
The decision curve analysis of the nomogram for non-dipperBP in hypertension patients in the training cohort.

In the model-development phase, a backward step-down selection process was performed according to the Akaike information criterion, using a threshold of *p* < 0.05 to establish a parsimonious (stepwise) model. This model was then used to formulate a nomogram in the training cohort (Figure 2). Cross-validation techniques were employed to evaluate the validity of the nomogram. The bootstrap method was used for the internal validation of the model, and an independent dataset was used for external validation. First, the training set, comprising 75% (n = 267) of the total randomized sample population, was extracted to build the nomogram. The remaining 25% (n = 89) was reserved as the validation set. Receiver operating characteristic (ROC) curves were plotted (Figure 3), and the area under the ROC curve (AUC) was calculated (Table 4). Sensitivity, specificity, positive predictive value (PPV), negative predictive value (NPV), positive likelihood ratio (PLR), and negative likelihood ratio (NLR) of the stepwise model are presented in Table 4. Decision curve analysis (Fitzgerald et al., 2015) was performed to determine the clinical usefulness of the model by calculating the net benefit, which is the proportion of individuals with true-positive results subtracted by the proportion of those who showed false-positive results, weighted by the relative hazard of false-positive and false-negative results (Figure 4). Moreover, ROC analyses were conducted to determine the optimal cutoff values for each risk factor. These values were defined as the points on the ROC curve where Youden’s index (sensitivity + specificity −1) was highest (Table 5). All statistical analyses were 2-tailed, with a *p-*value < 0.05 considered statistically significant. The analyses used R (http://www.R-project.org), EmpowerStats 4.0 (www.empowerstats.com, X&Y Solutions, Inc., Boston, MA), and SPSS 26.0.

**TABLE 4:** Prediction performance of the nomogram for estimating the prevelance of non-dipper blood pressure.

Items	Training group	Validation group
AUC	0.860	0.839
Sensitivity, %	0.785	0.746
Specificity, %	0.899	0.909
PPV, %	79.60	72.22
NPV, %	87.79	85.92
PLR	3.82	3.77
NLR	0.46	0.43

AUC, area under the receiver operating characteristic curve; PPV, positive predictive value; NPV, negative predictive value; PLR, positive likelihood ratio; NLR, negative likelihood ratio.

**TABLE 5:** Optimal cutoff values of related factors for non-dipper blood pressure.

Characteristic	Cutoff value	AUC	Sensitivity (%)	Specificity (%)
Age, year	41.50	0.749	75.76	69.57
sex	-	-	43.94	69.57
Hb, g/L	151.00	0.745	91.53	58.82
eGFR, ml/min/1.73m^2^	117.53	0.743	59.09	82.81
EF, %	64.50	0.680	88.52	47.37
Heart rate, times per minutes	75.00	0.701	54.55	78.26

AUC, area under the receiver operating characteristic curve, Hb hemoglobin; eGFR, estimated glomerular filtration rate; EF, ejection fraction.

## Results

### Baseline characteristics and related factors of non-dipper blood pressure

A total of 356 participants were included in this study, with 267 in the training group and 89 in the validation group, representing 75% and 25% of the total sample, respectively. Baseline comparisons showed no significant differences between the training and the validation groups (Table 1). Univariate analysis, with non-dipper blood pressure as the dependent variable, revealed substantial differences in age, sex, BMI, heart rate, Hb, eGFR, urea, TC, HDL-C, EF, PSQI score, daytime SBP, nighttime SBP, and nighttime DBP (Table 2). Multivariate regression analysis identified age, sex, Hb, eGFR, EF, and heart rate as having substantial differences (Table 3).

### Development and validation of the non-dipper blood pressure prediction nomogram

A nomogram model was developed to predict the probability of non-dipper blood pressure in patients with hypertension based on age, sex, Hb, eGFR, EF, and heart rate using the logistic binary regression analysis results. In this nomogram model, each factor is scored individually, and the total score, calculated by summing the individual scores of all indicators, determines the probability of non-dipper blood pressure occurrence.

The nomogram model demonstrated good predictive performance (Table 4). The area under the training group’s ROC curve (AUC) was 0.860, and that of the validation group was 0.839. The sensitivities of the training and validation groups were 78.50% and 74.60%, respectively. The specificity of the training and validation groups were 89.90% and 90.90%, respectively. The ROC curve obtained in this study is shown in Figure 3, and similar results were obtained in the training and validation groups.

### Decision curves for the nomogram predicting non-dipper blood pressure

**FIGURE 5:**
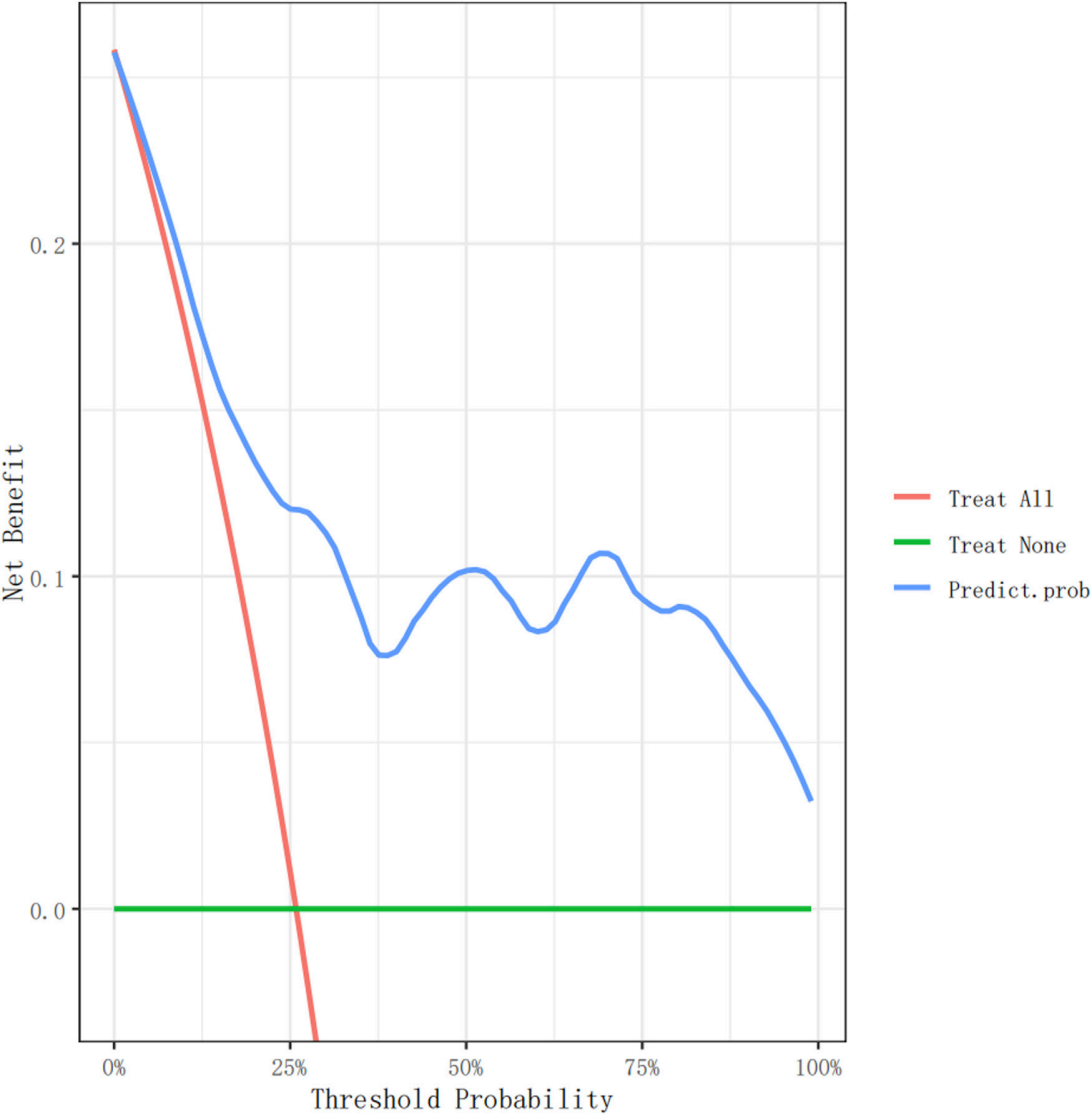
The decision curve analysis of the nomogram for non-dipper BP in hypepatients in the validation cohort.

Figures 4, 5 show the decision curve analysis (DCA) of the non-dipper blood pressure prediction model of patients with hypertension in the training and validation groups, respectively. The green line indicates that no participants were considered for non-dipper blood pressure, and the red line indicates that all participants were considered for non-dipper blood pressure. The blue line represents the clinical application of the nomogram predictive model. The farther the blue line is from the red and green lines, the more accurate the prediction of the nomogram model.

### Optimal cutoff values of related factors for non-dipper blood pressure prediction

Table 5 summarizes the optimal cutoff values of each related factor determined using the ROC analyses. The optimal cutoff values to predict non-dipper blood pressure for age, Hb, eGFR, EF, and heart rate are 41.50 years, 151.00 g/L, 117.53 mL/min/1.73 m^2^, 64.50%, and 75 beats per minute (bpm), respectively.

## Discussion

The blood pressure pattern of patients with hypertension is closely related to the prognosis of patients. Yang et al.'s study showed that the reduced rate of blood pressure drop at night was associated with a higher incidence of cerebrovascular diseases (Chen et al., 2021). Sun et al. have demonstrated that the magnitude of the decrease in blood pressure at night is negatively correlated with the risk of cerebral hemorrhage, and the incidence of minor blood vessel disease in the brain is increased in patients with non-dipper hypertension (Sun et al., 2014). Askın et al. (2021) showed that patients with non-dipper hypertension had an increased risk of cardiovascular disease and COPD and a decreased quality of life. Sun et al. (2016) showed that patients with non-dipper hypertension were independently associated with the occurrence of type 2 diabetes mellitus. Research by Kalaycı et al. (2019) demonstrated that non-dipper blood pressure correlates with a higher score on the age-adjusted Charlson Comorbidity Index (AACI), associated with increased patient mortality. Therefore, constructing a model to predict blood pressure patterns in patients with hypertension is critically essential.

Wang et al. (2021) highlighted the substantial number of untreated patients with hypertension in China, particularly among those from low-income families who face limited access to medical resources. Given the inadequate availability of ambulatory blood pressure monitoring devices in some parts of China, predicting blood pressure patterns using simple and accessible indicators becomes crucial. Patients with non-dipper hypertension experience poor prognosis. Therefore, our nomogram model facilitates early identification and intervention for these patients. Physicians can adjust the medication types and durations, and monitor medication adherence accordingly.

In this study, a nomogram model was developed to predict the occurrence of non-dipper blood pressure in patients with hypertension. The AUC of the training and validation groups were 0.860 and 0.839, respectively. Furthermore, DCA was employed to assess the clinical predictive efficacy of the nomogram model. Moreover, optimal cutoff values were identified for each factor associated with non-dipper blood pressure patterns in patients with hypertension. This model offers a straightforward and efficient means to identify non-dipper blood pressure in clinical settings.

Currently, research on predicting blood pressure patterns is limited. To the best of our knowledge, only Cortes-Rios et al. have developed a mathematical prediction model for blood pressure patterns (Cortés-Ríos and Rodriguez-Fernandez, 2020). Their model used calculus equations to predict changes in patients’ blood pressure circadian rhythms, incorporating independent variables such as norepinephrine (NE), physical activity, and glycemia. However, their findings were based on a review of literature data and not direct patient observations. Moreover, their mathematical model is complex and not straightforward for clinical application in judging patients’ blood pressure patterns simply and efficiently. In contrast, our study constructed a nomogram model using direct patient-derived data, encompassing training and validation cohorts. This approach enhances clinical applicability by offering a more efficient and simplified method.

Our results show that age, heart rate, sex, Hb, eGFR, and EF are significant factors influencing blood pressure patterns, consistent with previous research. Age emerges as a crucial risk factor in the prognosis of patients with hypertension. Xintian Cai et al. identified age as a significant risk factor for developing type 2 diabetes mellitus within 5 years among patients with hypertension (Cai et al., 2020). Age profoundly affects blood pressure patterns. Our study showed that the average age in the dipper-blood pressure group was 39.48 ± 11.67 years, whereas, in the non-dipper blood pressure group, it was 50.45 ± 11.48 years, demonstrating a significant difference between the two groups (*p* < 0.001). Similarly, Chu et al. study reported average ages of 52.2 ± 11.8 years in the dipper group and 56.2 ± 10.9 years in the non-dipper group (*p* < 0.05) (Chu et al., 2023). The study by Biyik et al. (2019) revealed that the mean age of patients with hypertension in the non-dipper blood pressure group was 57.7 ± 15.8 years, whereas those in the dipper blood pressure group had a mean age of 47.5 ± 13.5 years. These findings are consistent with our results. The increased probability of a non-dipper blood pressure pattern with advancing age may be attributed to younger patients being more active during the day, leading to higher daytime blood pressure during ambulatory blood pressure monitoring (ABPM). Consequently, younger patients tend to exhibit a more pronounced nighttime blood pressure drop compared to older patients with hypertension (Kaul et al., 2019).

In our study, the cutoff value for the effect of heart rate on blood pressure patterns was 75 bpm. Heart rate is closely related to blood pressure patterns, and previous studies have shown that heart rate variability impacts these patterns (Yan et al., 2020; Alp et al., 2021). However, the specific influence of average heart rate on blood pressure patterns was not determined in those studies, possibly due to their small sample sizes. The heart rate measured in our study was the resting heart rate of patients in the supine position during the day. Studies have shown that patients with a low heart rate are frequently associated with increased parasympathetic nerve activity (O’Neal et al., 2015; Arita et al., 1996), resulting in lower daytime blood pressure and, consequently, smaller nighttime blood pressure drop. Moreover, older patients generally have slower heart rates and an increased degree of arteriosclerosis, which may also be closely related to the occurrence of non-dipper blood pressure. However, the relationship between heart rate and blood pressure pattern requires further exploration.

Our study demonstrates that Hb levels affect blood pressure patterns, with a cutoff value of 151 g/L. Research indicates that patients with anemia have an increased risk of ischemic peripheral vascular disease, increased arteriosclerosis, and reduced vascular compliance (Vega et al., 2011; Esteban and Hernández-Rodríguez, 2022), which may contribute to non-dipper blood pressure rhythms in these patients.

Sex was identified as a predictor of non-dipper blood pressure in our study, with women with hypertension being more likely to have a non-dipper blood pressure pattern. However, the studies by Chavalit et al. (sample size: 208 cases) and Tsutomu et al. (sample size: 154) found that sex did not significantly affect blood pressure patterns (Chotruangnapa et al., 2021; Koike et al., 2023), which contradicts our findings. The limited number of studies on the relationship between sex and non-dipper blood pressure and its mechanisms suggests that more extensive sample-size studies are needed. David A Jaques et al. showed that systolic dipping status was positively associated with eGFR, with the dipper group having a higher eGFR than the non-dipper group, consistent with our findings (Jaques et al., 2021). Ciaran J McMullan et al. found that a 10% higher nocturnal dipping was significantly associated with a decreased risk of incident chronic kidney disease (CKD), indicating that loss of nocturnal blood pressure dipping may promote the decline in GFR and increase the risk of CKD development (McMullan et al., 2015). Our study similarly found that as eGFR increased, the probability of patients with non-dipper blood pressure decreased, revealing a strong relationship between reduced kidney function and the non-dipper blood pressure pattern. Gregory et al. reported a higher proportion of non-dipper blood pressure in the anemic group (Vyssoulis et al., 2011), consistent with our results. Moreover, our study found the cutoff value for the influence of ejection fraction on blood pressure patterns to be 64.5%. Previous studies have confirmed the relationship between heart failure and non-dipper blood pressure, indicating that non-dipper blood pressure is a predictor of heart failure (van de Borne et al., 1992; Ueda et al., 2019). Tigen et al. demonstrated that left ventricular systolic and diastolic functions are decreased in patients with non-dipper blood pressure (Tigen et al., 2009), consistent with our results. Qin et al. found a close association between left ventricular hypertrophy and the weakening or disappearance of the circadian rhythm of blood pressure (Ma et al., 2024). Our study suggests that ejection fraction can be used as a predictor of non-dipper blood pressure. The reasons may be as follows: the continuous increase in left ventricular afterload from non-dipper blood pressure prevents nighttime blood pressure reduction. Initially, this condition may cause compensatory myocardial thickening, eventually leading to a decline in diastolic function (Wei et al., 2014). Furthermore, patients with non-dipper blood pressure have an increased risk of sleep apnea at night (Salman et al., 2020). Sleep apnea is known to cause sympathetic overexcitation and myocardial tissue ischemia and hypoxia, which can further contribute to decreased myocardial diastolic function.

The advantages of this study include 1) the development of a model that can easily and quickly determine the blood pressure pattern of patients, improving clinical efficiency, and 2) the identification of cutoff values for each influencing factor. However, this study has limitations. Other indicators may influence blood pressure patterns; further investigation is needed to understand all factors contributing to blood pressure patterns fully. Antonio et al. found that the personality characteristics of patients with hypertension are significantly related to their blood pressure type, with reduced nighttime blood pressure dipping associated with antagonism and impulsivity-related traits, and not with measures of emotional vulnerability (Terracciano et al., 2014). Due to the time-consuming nature and inconvenience of personality trait assessments in clinical settings, our study did not collect this data. Although our prediction model does not include these factors, its prediction performance remains satisfactory. Another limitation of our study is the compromised generalizability of the results. Since this study only collected data from patients with hypertension in Shandong province, China, our findings may not apply to other ethnic groups. Future multi-center studies on blood pressure patterns are needed to address this issue.

In summary, our nomogram is a simple, plausible, affordable, and widely implementable tool to predict the blood pressure pattern of Chinese patients with hypertension. Our study provides valuable insights for determining the prognosis of patients with hypertension.

## Data Availability

The raw data supporting the conclusion of this article will be made available by the authors, without undue reservation.
