# Supplementary material for: Prediction of non-dipper blood pressure pattern in Chinese patients with hypertension using a nomogram model
Source: Front Physiol. 2024 Jul 24;15:1309212. doi: 10.3389/fphys.2024.1309212 (PMC11303159; doi:10.3389/fphys.2024.1309212)
Supplement: Supplementary file 1 [file Table1.docx]

**Table 1 Characteristics of the training and validation groups**

| **Characteristic** | **Training group**  **(n=267)** | **Validation group**  **(n=89)** | ***P* value** |
| --- | --- | --- | --- |
| Age, years | 47.62 ± 12.48 | 47.58 ± 12.53 | 0.979 |
| Sex, n (%) |  |  | 0.885 |
| Male | 159 (59.55%) | 54 (60.67%) |  |
| Famale | 108 (40.45%) | 35 (39.33%) |  |
| CHD, n (%) |  |  | 0.764 |
| 0 | 228 (85.39%) | 77 (86.51%) |  |
| 1 | 39 (14.61%) | 12 (13.49%) |  |
| Heart failure, n (%) |  |  | 0.856 |
| 0 | 263 (98.50%) | 88 (98.88%) |  |
| 1 | 4 (1.50%) | 1 (1.12%) |  |
| Diabetes, n (%) |  |  | 0.890 |
| 0 | 210 (78.65%) | 71 (79.78%) |  |
| 1 | 57 (21.35%) | 18 (20.22%) |  |
| BMI, kg/m^2^ | 26.28 ± 4.06 | 26.19 ± 3.98 | 0.856 |
| Heart rate, times per minutes | 75.96 ± 13.83 | 76.12 ± 13.11 | 0.904 |
| Hb, g/l | 139.71 ± 15.22 | 139.34 ± 15.01 | 0.842 |
| PLR | 139.93 ± 45.49 | 131.72 ± 36.92 | 0.144 |
| NLR | 0.36 ± 0.15 | 0.33 ± 0.99 | 0.100 |
| RDW,fl | 0.12 ± 0.01 | 0.12 ± 0.01 | 0.862 |
| Creatinine, µmol/l | 67.41 ± 27.18 | 69.20 ± 31.60 | 0.611 |
| eGFR, ml/min/1.73m^2^ | 105.19 ± 18.99 | 104.03 ± 21.27 | 0.633 |
| Urea, mmol/l | 5.49 ± 1.88 | 5.51 ± 1.79 | 0.930 |
| Uric acid, µmol/l | 339.66 ± 104.95 | 336.40 ± 103.21 | 0.799 |
| TG, mmol/l | 1.78 ± 1.34 | 1.79 ± 1.31 | 0.951 |
| TC, mmol/l | 4.74 ± 1.31 | 4.76 ± 1.33 | 0.901 |
| HDLC, mmol/l | 1.24 ± 0.30 | 1.24 ± 0.28 | 0.994 |
| LDLC, mmol/l | 3.00 ± 1.02 | 3.01 ± 1.01 | 0.936 |
| HCY, µmol/l | 11.06 ± 4.19 | 10.89 ± 4.12 | 0.739 |
| EF, % | 61.80 ± 3.53 | 60.96 ± 3.66 | 0.054 |
| Midicine |  |  |  |
| ARNI | 129 (48.3%) | 51 (57.3%) | 0.089 |
| β-block | 79 (29.6%) | 21 (23.6%) | 0.170 |
| CCB | 122 (45.7%) | 33(37.1%) | 0.097 |
| Diuretic | 27 (10.1%) | 4 (4.4%) | 0.069 |
| ARB/ACEI | 42 (15.7%) | 8 (9.0%) | 0.076 |
| Arotinolol | 18 (6.7%) | 7 (7.9%) | 0.439 |
| PSQI | 7.70 ± 3.71 | 7.72 ± 3.74 | 0.965 |
| OH | 45 (16.9%) | 15 (16.7%) | 0.550 |
| Daytime SBP, mmHg | 132.94 ± 13.45 | 133.17 ± 13.53 | 0.892 |
| Nighttime SBP, mmHg | 127.06 ± 15.94 | 127.49 ± 16.27 | 0.826 |
| Daytime DBP, mmHg | 86.50 ± 10.77 | 86.47 ± 10.73 | 0.977 |
| Nighttime DBP, mmHg | 82.58 ± 12.05 | 82.78 ± 12.11 | 0.893 |
| 24-MEANSBP, mmHg | 131.83 ± 13.14 | 132.03 ± 13.10 | 0.901 |
| 24-MEANDBP, mmHg | 85.62 ± 10.75 | 85.70 ± 10.81 | 0.952 |

CHD coronary heart disease, BMI body mass index, Hb hemoglobin, PLR platelet-to-lymphocyte ratio, NLR neutrophil-to-lymphocyte ratio, RDW red cell distribution width, eGFR estimated glomerular filtration rate, TG triglyceride, TC total cholesterol, HDL-C high density lipoprotein cholesterol, LDL-C low density lipoprotein cholesterol, Hcy homocysteine, EF ejection fraction, PSQI pittsburgh sleep quality index, OH orthostatic hypotension, Daytime SBP daytime systolic blood pressure, Nighttime SBP nighttime systolic blood pressure, Daytime DBP daytime diastole blood pressure, Nighttime DBP nighttime diastole blood pressure, 24-h mean SBP 24-hour mean systolic blood pressure, 24-h mean DBP 24-hour mean diastole blood pressure.

**Table 2 Baseline characteristics according to the prevalence of non-dipper blood pressure and the univariate logistic regression analysis in the training group (n=267)**

| **Characteristic** | **Prevelance of non-dipper BP** | | **Univariate**  **logistic regression analysis** | |
| --- | --- | --- | --- | --- |
|  | **Non-dipper**  **(n=198)** | **Dipper BP**  **(n=69)** | **OR** | ***P*** |
| Age, year | 50.45 ± 11.48 | 39.48 ± 11.67 | 1.09 (1.06, 1.12) | <0.001 |
| Sex, n (%) |  |  | 1.79 (1.08, 2.97) | 0.024 |
| Male | 111 (56.06%) | 48 (69.57%) |  |  |
| Famale | 87 (43.94%) | 21 (30.43%) |  |  |
| CHD, n (%) |  |  | 2.10 (0.95, 4.65) | 0.067 |
| 0 | 165 (83.33%) | 63 (91.30%) |  |  |
| 1 | 33 (16.67%) | 6 (8.70%) |  |  |
| Heart failure, n (%) |  |  | 0.18 (-0.10, 0.45) | 0.771 |
| 0 | 195 (98.48%) | 68 (98.55%) |  |  |
| 1 | 3 (1.52%) | 1 (1.45%) |  |  |
| Diabetes, n (%) |  |  | 1.40 (0.76, 2.57) | 0.283 |
| 0 | 153 (77.27%) | 57 (82.61%) |  |  |
| 1 | 45 (22.73%) | 12 (17.39%) |  |  |
| BMI, kg/m^2^ | 25.55 ± 3.54 | 28.36 ± 4.64 | 0.83 (0.78, 0.89) | <0.001 |
| Heart rate, times per minutes | 73.65 ± 13.50 | 83.04 ± 12.27 | 0.95 (0.93, 0.97) | <0.001 |
| Hb, g/l | 136.93 ± 14.57 | 149.35 ± 13.04 | 0.93 (0.90, 0.95) | <0.001 |
| PLR | 140.08 ± 47.19 | 137.82 ± 27.13 | 1.00 (0.99,1.01) | 0.818 |
| NLR | 0.37 ± 0.16 | 0.33 ± 0.12 | 0.24 (0.01, 4.70) | 0.354 |
| RDW,fl | 0.12 ± 0.01 | 0.12 ± 0.01 | inf.(0.00, inf.) | 0.276 |
| Creatinine, µmol/l | 66.92 ± 26.28 | 60.84 ± 11.56 | 0.99 (0.97, 1.01) | 0.174 |
| eGFR, ml/min/1.73m^2^ | 100.43 ± 19.42 | 116.48 ± 13.37 | 1.07 (1.04, 1.11) | <0.001 |
| Urea, mmol/l | 5.67 ± 2.00 | 4.84 ± 1.11 | 1.49 (1.17, 1.90) | 0.001 |
| Uric acid, µmol/l | 334.72 ± 101.20 | 357.56 ± 114.39 | 1.00 (1.00, 1.00) | 0.122 |
| TG, mmol/l | 1.76 ± 1.42 | 1.84 ± 0.94 | 0.96 (0.78, 1.16) | 0.648 |
| TC, mmol/l | 4.83 ± 1.34 | 4.45 ± 1.10 | 1.27 (1.01, 1.59) | 0.040 |
| HDLC, mmol/l | 1.28 ± 0.32 | 1.12 ± 0.14 | 12.45 (3.04, 51.07) | <0.001 |
| LDLC, mmol/l | 3.04 ± 1.04 | 2.89 ± 0.94 | 1.15 (0.87, 1.52) | 0.324 |
| HCY, µmol/l | 10.86 ± 3.33 | 11.67 ± 5.99 | 0.96 (0.91, 1.01) | 0.127 |
| EF, % | 61.25 ± 3.45 | 63.58 ± 3.10 | 0.78 (0.70, 0.86) | <0.001 |
| Midicine |  |  |  |  |
| ARNI | 98 (49.5%) | 31 (44.9%) | 0.83 (0.48, 1.44) | 0.304 |
| β-block | 60 (30.3%) | 19 (27.5%) | 0.87 (0.47, 1.61) | 0.393 |
| CCB | 88 (44.4%) | 35 (50.7%) | 1.29 (0.74, 2.23) | 0.223 |
| Diuretic | 18 (9.1%) | 9 (13.0%) | 0.65 (0.31, 1.88) | 0.122 |
| ARB/ACEI | 29 (14.6%) | 14 (20.3%) | 1.48 (0.73, 3.01) | 0.181 |
| Arotinolol | 15 (7.6%) | 4 (5.8%) | 0.75 (0.24, 2.34) | 0.427 |
| PSQI | 8.18 ± 3.91 | 6.30 ± 2.54 | 1.19 (1.10, 1.30) | <0.001 |
| OH | 36 (18.2%) | 9 (13.0%) | 0.68 (0.31, 1.49) | 0.216 |
| Daytime SBP, mmHg | 131.74 ± 13.24 | 136.42 ± 13.73 | 1.03 (1.01, 1.05) | 0.014 |
| Nighttime SBP, mmHg | 130.58 ± 15.80 | 116.91 ± 12.48 | 0.93 (0.91, 0.96) | <0.001 |
| Daytime DBP, mmHg | 86.11 ± 10.51 | 87.84 ± 11.37 | 1.02 (0.99, 1.05) | 0.248 |
| Nighttime DBP, mmHg | 85.04 ± 11.31 | 75.80 ± 11.77 | 0.93 (0.91, 0.96) | <0.001 |
| 24-MEANSBP, mmHg | 131.53 ± 13.16 | 132.70 ± 12.89 | 0.99 (0.98, 1.01) | 0.462 |
| 24-MEANDBP, mmHg | 85.71 ± 10.40 | 85.35 ± 11.58 | 1.00 (0.98, 1.03) | 0.778 |

CHD coronary heart disease, BMI body mass index, Hb hemoglobin, PLR platelet-to-lymphocyte ratio, NLR neutrophil-to-lymphocyte ratio, RDW red cell distribution width, eGFR estimated glomerular filtration rate, TG triglyceride, TC total cholesterol, HDL-C high density lipoprotein cholesterol, LDL-C low density lipoprotein cholesterol, Hcy homocysteine, EF ejection fraction, PSQI pittsburgh sleep quality index, OH orthostatic hypotension, Daytime SBP daytime systolic blood pressure, Nighttime SBP nighttime systolic blood pressure, Daytime DBP daytime diastole blood pressure, Nighttime DBP nighttime diastole blood pressure, 24-h mean SBP 24-hour mean systolic blood pressure, 24-h mean DBP 24-hour mean diastole blood pressure.

**Table 3 Multivariate logistic regression analysis for related factors associated non-dipper blood pressure in the training cohort (n=267)**

| **Variable** | ***β*** | ***SE*** | **Wald *χ*^2^** | ***P*** | ***OR*** |
| --- | --- | --- | --- | --- | --- |
| Age, year | 0.116 | 0.038 | 9.442 | 0.002 | 1.123 |
| Sex, n (%) |  |  |  | ＜0.001 | 0.005 |
| Male | -5.372 | 0.972 | 30.579 |  |  |
| Female | Ref |  |  |  |  |
| Hb, g/l | -0.201 | 0.035 | 33.460 | ＜0.001 | 0.818 |
| eGFR, ml/min/1.73m^2^ | -0.051 | 0.023 | 4.736 | 0.030 | 0.951 |
| EF, % | -0.496 | 0.130 | 14.540 | ＜0.001 | 0.609 |
| Heart rate, times per minutes | -0.050 | 0.021 | 5.657 | 0.017 | 0.951 |

Hb hemoglobin, eGFR estimated glomerular filtration rate, EF ejection fraction.

**Table 4 Prediction performance of the nomogram for estimating the prevelance of non-dipper blood pressure**

| **Items** | **Training group** | **Validation group** |
| --- | --- | --- |
| AUC | 0.860 | 0.839 |
| Sensitivity, % | 0.785 | 0.746 |
| Specificity, % | 0.899 | 0.909 |
| PPV, % | 79.60 | 72.22 |
| NPV, % | 87.79 | 85.92 |
| PLR | 3.82 | 3.77 |
| NLR | 0.46 | 0.43 |

AUC area under the receiver operating characteristic curve, PPV positive predictive value, NPV negative predictive value, PLR positive likelihood ratio, NLR negative likelihood ratio.

**Table 5 Optimal cutoff values of related factors for non-dipper blood pressure**

| **Characteristic** | **Cutoff value** | **AUC** | **Sensitivity (%)** | **Specificity (%)** |
| --- | --- | --- | --- | --- |
| Age, year | 41.50 | 0.749 | 75.76 | 69.57 |
| sex | - | - | 43.94 | 69.57 |
| Hb, g/l | 151.00 | 0.745 | 91.53 | 58.82 |
| eGFR, ml/min/1.73m^2^ | 117.53 | 0.743 | 59.09 | 82.81 |
| EF, % | 64.50 | 0.680 | 88.52 | 47.37 |
| Heart rate, times per minutes | 75.00 | 0.701 | 54.55 | 78.26 |

AUC area under the receiver operating characteristic curve, Hb hemoglobin, eGFR estimated glomerular filtration rate, EF ejection fraction.
